# Assessing progression limits in different grades of keratoconus from a novel perspective: precision of measurements of the corneal epithelium

**DOI:** 10.1186/s40662-023-00368-9

**Published:** 2024-01-02

**Authors:** Rui Ning, Yiran Wang, Zhenyu Xu, Ingemar Gustafsson, Jiawei Li, Giacomo Savini, Domenico Schiano-Lomoriello, Yichen Xiao, Aodong Chen, Xiaoying Wang, Xingtao Zhou, Jinhai Huang

**Affiliations:** 1 Eye Institute and Department of Ophthalmology and Vision Science, Institute for Medical and Engineering Innovation, Eye and ENT Hospital, Fudan University, N No.19 Baoqing Road, Xuhui District, Shanghai, 200031 China; 2 Shanghai Research Center of Ophthalmology and Optometry, Shanghai, China; 3 Department of Clinical Sciences, Ophthalmology, Lund University, Skåne University Hospital, Malmö, Sweden; 4 IRCCS Bietti Foundation, Rome, Italy; 5 NHC Key Laboratory of Myopia (Fudan University), Key Laboratory of Myopia, Chinese Academy of Medical Sciences, Shanghai, China

**Keywords:** Keratoconus, Corneal epithelium thickness, Precision, Progression limits

## Abstract

**Background:**

To assess repeatability and reproducibility of corneal epithelium thickness (ET) measured by a spectral-domain optical coherence tomographer (SD-OCT)/Placido topographer (MS-39, CSO, Florence, Italy) in keratoconus (KC) population at different stages, as well as to determine the progression limits for evaluating KC progression.

**Methods:**

A total of 149 eyes were enrolled in this study, with 29 eyes in the forme fruste keratoconus (FFKC) group, 34 eyes in the mild KC group, 40 eyes in the moderate KC group, and 46 eyes in the severe KC group. Employing the within-subject standard deviation (S_w_), test-retest variability (TRT), coefficient of variation (CoV), and intraclass correlation coefficient (ICC) to evaluate intraoperator repeatability and interoperator reproducibility.

**Results:**

The repeatability and reproducibility of MS-39 in patients with KC were acceptable, according to ICC values ranging from 0.732 to 0.954. However, patients with more severe KC and progressive peripheralization of the measurement points had higher TRTs but a thinning trend. The current study tended to set the cut-off values of mild KC, moderate KC, and severe KC to 4.9 µm, 5.2 µm, and 7.4 µm for thinnest epithelium thickness (TET). When differences between follow-ups are higher than those values, progression of the disease is possible. As for center epithelium thickness (CET), cut-off values for mild KC, moderate KC, and severe KC should be 2.8 µm, 4.4 µm, and 5.3 µm. This might be useful in the follow-up and diagnosis of keratoconus.

**Conclusions:**

This study demonstrated that the precision of MS-39 was reduced in measuring more severe KC patients and more peripheral corneal points. In determining disease progression, values should be differentiated between disease-related real changes and measurement inaccuracies. Due to the large difference in ET measured by MS-39 between various stages of disease progression, it is necessary to accurately grade KC patients to avoid errors in KC clinical decision-making.

**Supplementary Information:**

The online version contains supplementary material available at 10.1186/s40662-023-00368-9.

## Background

Keratoconus (KC) is a progressive disease that can cause severe visual impairment. However, corneal cross-linking can delay or stop the disease progression and thus avoid deteriorating visual acuity and the need for corneal transplantation surgery [1–4]. The indication for corneal cross-linking is commonly progressive keratoconus [2, 5–7]. It has been suggested that an increased curvature of the anterior or posterior corneal surfaces and a thinning of the cornea are suggestive of keratoconus disease progression [2]. The most commonly used parameter is maximum keratometry value (K_max_) but a recent survey showed that the ABCD Progression Display is also frequently used [8, 9]. However, other parameters have also been suggested for diagnosing progressive keratoconus. For example, epithelial distribution of keratoconus was proven to aid in early diagnosis and monitoring of disease progression following corneal cross-linking [10, 11]. The epithelium is the outermost structure of the cornea and has a strong ability to remodel to provide a smooth optical surface [12]. In keratoconus, epithelial remodeling occurs to reduce stromal irregularity [13, 14]. Reinstein et al. have suggested that a particular change of the epithelium in patients with keratoconus is an “epithelial doughnut pattern” [15–17]. It has been proposed that these changes can be used to evaluate the progression of the keratoconus disease because the epithelium can alter in response to the keratoconus disease [12, 18, 19].

In this study, an optical coherence tomographer (MS-39, CSO, Florence, Italy) was used to obtain measurements of the epithelial thickness (ET). The repeatability and reproducibility were evaluated to determine whether a change in a parameter's magnitude is the result of measurement error or a genuine change when evaluating new diagnostic parameters [20, 21]. Furthermore, as prior investigations have shown an association between the repeatability of the measurements and the keratoconus disease severity, the repeatability was assessed in patients with varying degrees of keratoconus [22, 23]. Here, we provided cut-off criteria for the assessment of progressive patients with different degrees of keratoconus.

### Methods

## Patients

This prospective study followed the principles of the Declaration of Helsinki and was approved by the Ethics Committee of the Eye and ENT Hospital Review Board of Fudan University (2021174). All patients were informed about the objectives and procedures of the study, and all participants provided written informed consent.

Patients with the diagnosis of KC were enrolled in this study. In subjects diagnosed with bilateral keratoconus, the eye for the study was selected randomly. The inclusion criteria for patients with clinical keratoconus were history of vision loss, at least one of the biomicroscopic signs (Vogt’s striae, Fleischer ring, or focal stromal thinning), characteristic keratoconus signs on corneal tomography, such as skewed asymmetric bow-tie and inferior steepening. The above selected eyes were further graded as mild KC, moderate KC and severe KC. Additionally, patients with forme fruste keratoconus (FFKC) were defined as having the following characteristics: keratoconus in the fellow eye, normal-appearing on slit-lamp biomicroscopy, and normal topography: (i) K_max_ < 47.2 diopters, inferior-superior difference value (I-S value) at 6 mm ≤ 1.4; (ii) Keratoconus percentage index (KISA% index) < 60% [24]. Exclusion criteria were: active ocular disease or trauma, corneal hydrops and extensive corneal scarring, dry eye, previous ocular surgery (including corneal cross-linking surgery), and a history of wearing contact lenses (for soft contact lenses less than two weeks, rigid contact lenses less than four weeks). Pregnant women, atopic patients with a history of herpes, and trisomy 21 were also excluded in this study. The severity of keratoconus was divided into three groups using the Pentacam HR (OCULUS, Wetzlar, Germany) topographical keratoconus classification (TKC), which is based on the anterior corneal surface parameters, such as index of surface variance (ISV), keratoconus index (KI), smallest radius (Rmin). The following were the subgroups: TKC 1, 1–2 for mild KC; TKC 2, 2–3 for moderate KC; TKC 3, 3–4 for severe KC [25, 26].

### Instruments

The MS-39 based SD-OCT and Placido-disk corneal tomographer utilizes an 845 nm super luminescent light-emitting diode (SLED) light source and generates high-definition images with an axial resolution of 3.6 μm in tissue and transversal resolution of 35 μm in air. Furthermore, it scans 25 meridians on a 16 mm transversal field. The corneal anterior and posterior surfaces can be measured at 31,232 and 25,600 points, respectively. The ET measured by MS-39 is the distance between the tear film layer and Bowman’s layer.

### Procedures

All patients were measured three times consecutively by two experienced operators using the MS-39 tomographer. To minimize diurnal change and eye effects due to sleep and eye closure, measurements were taken in a dim room between 9 a.m. and 5 p.m. after volunteers had been awake for at least two hours. To ensure that the tear film was spread out evenly, patients were instructed to blink immediately before each measurement and to keep their eyes open during the measurement. During the testing, no eye drops were used. The entire process took less than 15 min. All operating procedures are strictly followed to the manufacturer’s specifications. The examination was included in the analysis if the quality specification was satisfactory. If not, the procedure was repeated.

ET measurements are analyzed, including center epithelium thickness (CET) and thinnest epithelium thickness (TET). Meanwhile, the eight remaining points of ET are measured at superior, inferior, nasal, and temporal distances of 1 mm and 3 mm from the apex of the cornea, which appears as abbreviations: S_1_, S_3_, I_1_, I_3_, N_1_, N_3_, T_1_, T_3_.

### Statistical analysis

SPSS (version 21.0; IBM Corp., Armonk, NY, USA) and GraphPad Prism 9.00 were used for statistical analysis (GraphPad Software, USA). The Kolmogorov–Smirnov test was used to determine whether the data were normally distributed (*P* > 0.05). For multiple comparisons between groups, statistical significance was determined using one-way analysis of variance (ANOVA) and Tukey’s post-hoc test. The data were presented as mean ± standard deviation (SD). Within-subject standard deviation (S_w_), test-retest variability (TRT), coefficient of variation (CoV), and intraclass correlation coefficient (ICC) were used to assess intraoperator repeatability and interoperator reproducibility. Repeatability was independently assessed for both operators. *P* values less than 0.05 were considered statistically significant. The square root of variance in a subject is S_w_. The TRT was 2.77S_w_, indicating the 95% distribution range of the difference from multiple observations. Lower values of S_w_ and TRT values represent better precision. CoV is the ratio of S_w_ to the total mean values. The smaller the values of S_w_, TRT, and CoV, the better their repeatability [21]. ICCs can be calculated through ANOVA, which is a reliability coefficient. The closer the value is to 1, the higher the reliability.

### Sample size calculation

Previous studies have demonstrated that small sample sizes do not provide sufficient confidence in the results of repeatability studies [27]. Specifically, current study design involves three repeated measures and a sample size of more than 96, the confidence in the estimate is 0.1.

## Results

A total of 149 eyes of 149 patients were included in this study, with a mean age of 24.67 ± 6.59 years (range: 12 to 43 years). Among them, there were 29 eyes in the FFKC group, 34 eyes in the mild KC group, 40 eyes in the moderate KC group and 46 eyes in the severe KC group.

**Fig. 1:**
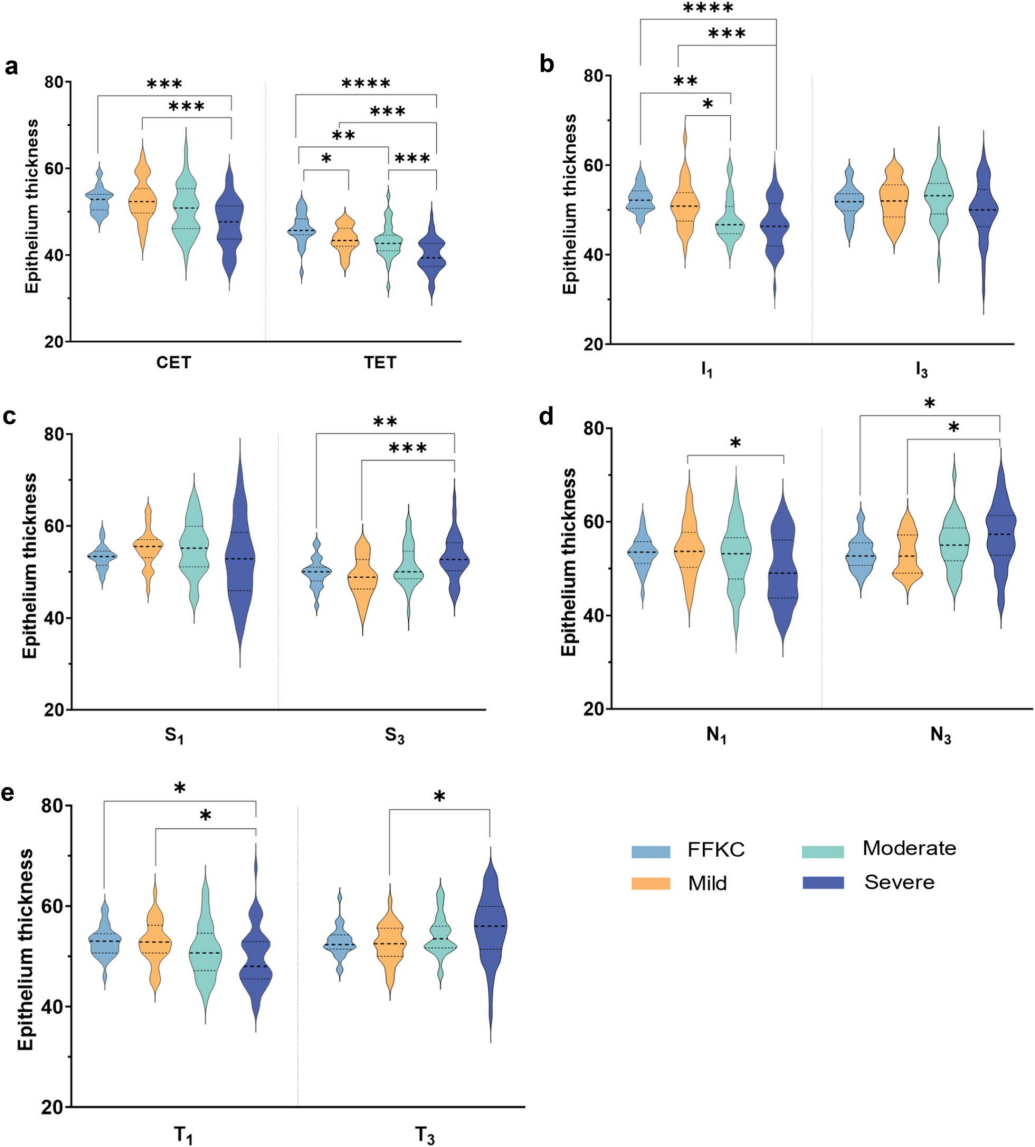
The Violin plots of epithelium thickness obtained with the spectral-domain optical coherence tomographer (SD-OCT)/Placido device at difference stages of keratoconus groups. **a** Epithelium thickness of central epithelium thickness (CET) and thinnest epithelium thickness (TET); **b** Epithelium thickness of I_1_ and I_3_; **c** Epithelium thickness of S_1_ and S_3_; **d** Epithelium thickness of N_1_ and N_3_; **e** Epithelium thickness of T_1_ and T_3_. I_1_ (I_3_), corneal apex inferior at 1 mm (3 mm); S_1_ (S_3_), corneal apex superior at 1 mm (3 mm); N_1_ (N_3_), corneal apex nasal at 1 mm (3 mm); T_1_ (T_3_), corneal apex temporal at 1 mm (3 mm); *P ≤ 0.05, **P ≤ 0.01, ***P ≤ 0.001, ****P ≤ 0.0001

The Violin plots in Fig. 1 show the ET values of each group at CET, TET, I_1_, I_3_, S_1_, S_3_, N_1_, N_3_, T_1_, and T_3_. The area represents the probability of distribution around a value. The top and bottom black dotted lines represent the interquartile range, while the middle black line is the median limbus.

### Intraobserver repeatability in the measurement of ET

**Fig. 2:**
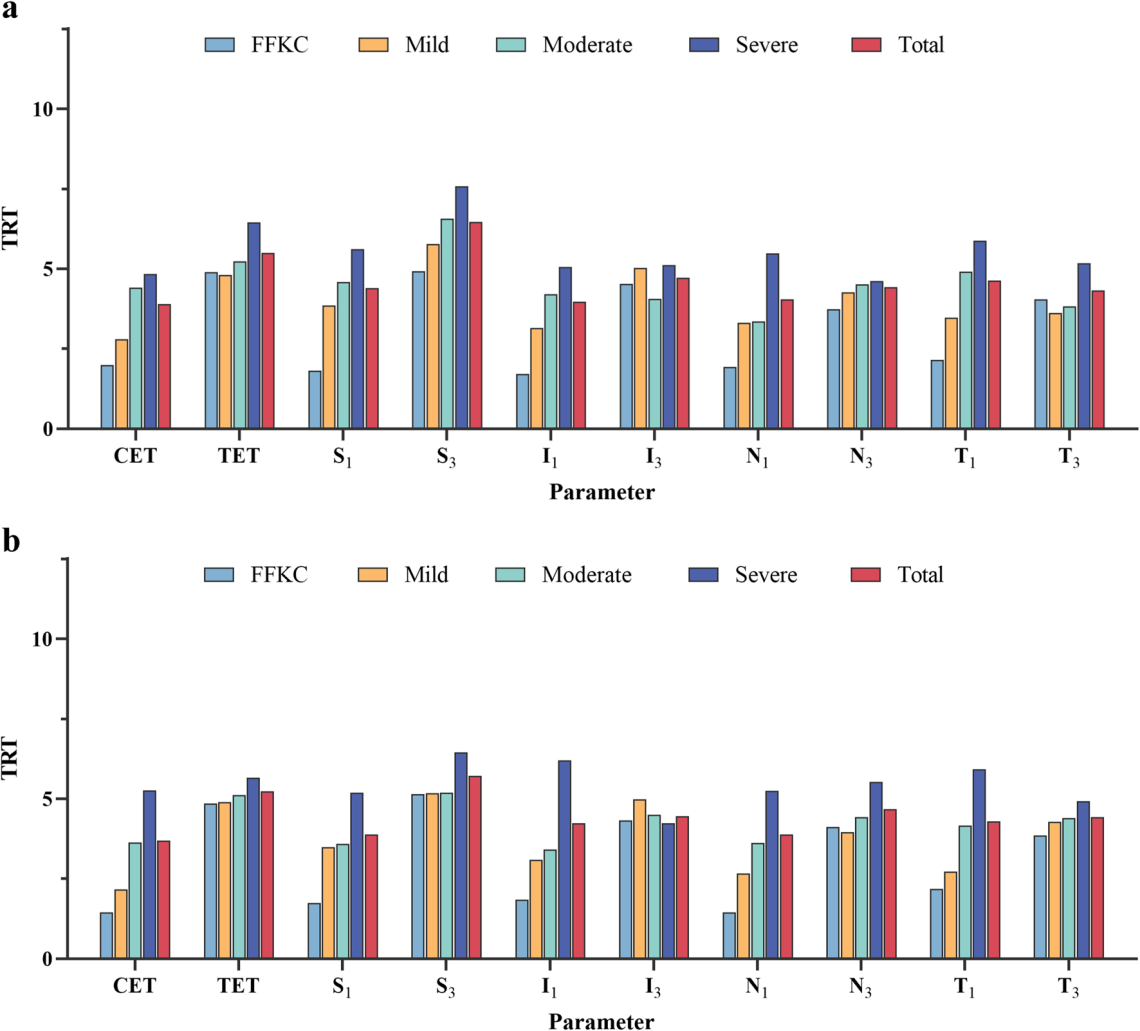
TRT values for repeatability at different stages of keratoconus measured by observer 1 (**a**) and observer 2 (**b**). TRT, test-retest repeatability (2.77 S_w_); CET, central epithelium thickness; TET, thinnest epithelium thickness; S_1_ (S_3_), corneal apex superior at 1 mm (3 mm); I_1_ (I_3_), corneal apex inferior at 1 mm (3 mm); N_1_ (N_3_), corneal apex nasal at 1 mm (3 mm); T_1_ (T_3_), corneal apex temporal at 1 mm (3 mm)

**Fig. 3:**
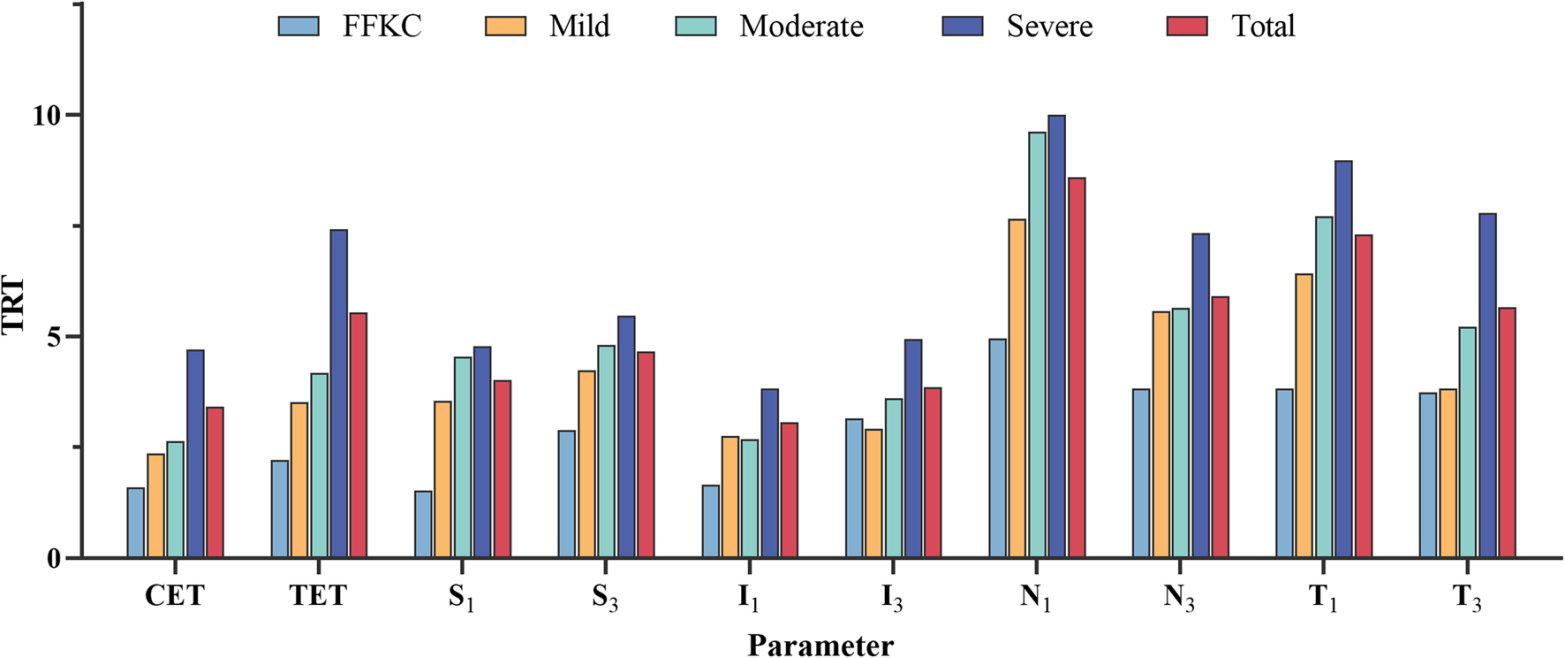
TRT values for reproducibility at different stages of keratoconus. TRT, test-retest repeatability (2.77 S_w_); CET, central epithelium thickness; TET, thinnest epithelium thickness; S_1_ (S_3_), corneal apex superior 1 mm (3 mm); I_1_ (I_3_), corneal apex inferior at 1 mm (3 mm); N_1_ (N_3_), corneal apex nasal at 1 mm (3 mm); T_1_ (T_3_), corneal apex temporal at 1 mm (3 mm)

Tables 1, 2, 3, 4, 5 show the repeatability of ET measurement in the FFKC group, mild KC group, moderate KC group, severe KC group, and total subjects, including CET, TET, S_1_, S_3_, I_1_, I_3_, N_1_, N_3_, T_1_, T_3_. In FFKC, mild KC, moderate KC, and total groups (Tables 1, 2, 3, 5), MS-39 shows excellent repeatability in two observers for CET, S_1_, I_1_, N_1_ and T_1_, with all ICCs ≥ 0.9 and TRT values range from 1.45 to 2.18 μm, 2.17 to 3.86 μm, 3.36 to 4.91 μm, 3.7 to 4.63 μm. The TRT values at TET, S_3_, I_3_, N_3_, and T_3_ fluctuate from 3.74 to 5.14 μm, 3.62 to 5.78 μm, 3.83 to 6.57 μm, 4.32 to 6.47 μm, respectively. As for the severe group (Table 4), values of TRT range from 5.06 to 6.21 μm, 4.24 to 7.58 μm for points measured at 1 mm and 3 mm separately. Figures 2 and 3 show the TRT repeatability values in varying degrees of KC patients at all locations as histograms.

**Table 1:** Intraoperator repeatability for epithelium thickness obtained using MS-39 in forme fruste keratoconus patients

Parameter	Observer	Mean ± SD (μm)	S_w_ (μm)	TRT (μm)	CoV (%)	ICC (95% CI)
CET	1st	52.62 ± 2.85	0.52	1.45	0.99	0.967 (0.939 to 0.983)
	2nd	52.68 ± 2.91	0.72	2.00	1.37	0.941 (0.893 to 0.970)
TET	1st	46.12 ± 3.59	1.75	4.85	3.79	0.795 (0.657 to 0.891)
	2nd	46.08 ± 3.57	1.77	4.90	3.84	0.789 (0.648 to 0.887)
S_1_	1st	53.24 ± 2.48	0.63	1.74	1.18	0.939 (0.889 to 0.969)
	2nd	53.40 ± 2.28	0.65	1.81	1.23	0.922 (0.860 to 0.960)
S_3_	1st	49.80 ± 3.19	1.86	5.14	3.73	0.723 (0.532 to 0.860)
	2nd	49.69 ± 3.04	1.78	4.92	3.58	0.721 (0.547 to 0.850)
I_1_	1st	52.38 ± 2.79	0.66	1.84	1.27	0.946 (0.901 to 0.972)
	2nd	52.79 ± 2.83	0.62	1.71	1.17	0.954 (0.916 to 0.977)
I_3_	1st	51.74 ± 3.51	1.56	4.32	3.01	0.826 (0.704 to 0.908)
	2nd	52.11 ± 3.09	1.64	4.53	3.14	0.764 (0.611 to 0.873)
N_1_	1st	53.30 ± 3.44	0.52	1.45	0.98	0.977 (0.958 to 0.989)
	2nd	54.31 ± 2.57	0.70	1.94	1.29	0.930 (0.873 to 0.964)
N_3_	1st	53.12 ± 3.46	1.49	4.12	2.80	0.836 (0.719 to 0.914)
	2nd	53.51 ± 2.91	1.35	3.74	2.52	0.812 (0.682 to 0.900)
T_1_	1st	52.90 ± 3.02	0.79	2.18	1.49	0.935 (0.883 to 0.967)
	2nd	52.26 ± 3.63	0.78	2.16	1.49	0.955 (0.919 to 0.978)
T_3_	1st	52.81 ± 2.93	1.39	3.86	2.64	0.804 (0.670 to 0.896)
	2nd	52.20 ± 3.18	1.46	4.05	2.80	0.814 (0.686 to 0.902)

*SD* = standard deviation; *S*_*w*_ = within-subject standard deviation; *TRT* = test-retest repeatability (2.77 S_w_); *CoV* = coefficient of variation; *ICC* = intraclass correlation coefficient; *CET* = central epithelium thickness; *TET* = thinnest epithelium thickness; *S*_*1*_ *(S*_*3*_*)* = corneal apex superior at 1 mm (3 mm); *I*_*1*_ *(I*_*3*_*)* = corneal apex inferior at 1 mm (3 mm); *N*_*1*_* (N*_*3*_*)* = corneal apex nasal at 1 mm (3 mm); *T*_*1*_ *(T*_*3*_*)* = corneal apex temporal at 1 mm (3 mm)

**Table 2:** Intraoperator repeatability for epithelium thickness obtained using MS-39 in mild keratoconus patients

Parameter	Observer	Mean ± SD (μm)	S_w_ (μm)	TRT (μm)	CoV (%)	ICC (95% CI)
CET	1st	52.40 ± 4.81	0.78	2.17	1.49	0.974 (0.953 to 0.987)
	2nd	52.59 ± 4.68	1.01	2.80	1.92	0.955 (0.920 to 0.976)
TET	1st	43.63 ± 2.85	1.76	4.89	4.04	0.695 (0.522 to 0.828)
	2nd	44.11 ± 3.29	1.73	4.80	3.93	0.767 (0.624 to 0.870)
S_1_	1st	55.08 ± 4.18	1.26	3.49	2.29	0.914 (0.850 to 0.955)
	2nd	54.65 ± 4.25	1.40	3.86	2.55	0.900 (0.827 to 0.946)
S_3_	1st	48.47 ± 4.17	1.87	5.18	3.86	0.823 (0.686 to 0.912)
	2nd	48.41 ± 4.01	2.09	5.78	4.31	0.770 (0.627 to 0.874)
I_1_	1st	50.44 ± 4.33	1.12	3.09	2.21	0.936 (0.887 to 0.967)
	2nd	50.58 ± 4.36	1.14	3.15	2.25	0.935 (0.886 to 0.966)
I_3_	1st	51.92 ± 4.44	1.80	4.98	3.46	0.852 (0.749 to 0.921)
	2nd	51.82 ± 4.33	1.81	5.02	3.50	0.843 (0.737 to 0.915)
N_1_	1st	53.61 ± 5.41	0.96	2.66	1.79	0.969 (0.944 to 0.984)
	2nd	54.82 ± 4.30	1.20	3.31	2.18	0.926 (0.871 to 0.961)
N_3_	1st	52.81 ± 4.12	1.43	3.95	2.70	0.889 (0.809 to 0.941)
	2nd	53.47 ± 4.08	1.54	4.27	2.88	0.870 (0.780 to 0.930)
T_1_	1st	52.51 ± 4.23	0.98	2.72	1.87	0.948 (0.907 to 0.973)
	2nd	51.34 ± 4.65	1.25	3.47	2.44	0.931 (0.879 to 0.964)
T_3_	1st	52.19 ± 3.69	1.55	4.28	2.96	0.843 (0.735 to 0.916)
	2nd	51.80 ± 4.07	1.31	3.62	2.52	0.903 (0.833 to 0.949)

*SD* = standard deviation; *S*_*w*_ = within-subject standard deviation; *TRT* = test-retest repeatability (2.77 S_w_); *CoV *= coefficient of variation; *ICC* = intraclass correlation coefficient; *CET* = central epithelium thickness; *TET* = thinnest epithelium thickness; *S*_*1*_ *(S*_*3*_*)* = corneal apex superior at 1 mm (3 mm); *I*_*1*_ *(I*_*3*_*)* = corneal apex inferior at 1 mm (3 mm); *N*_*1*_ *(N*_*3*_*)* = corneal apex nasal at 1 mm (3 mm); *T*_*1*_ *(T*_*3*_*)* = corneal apex temporal at 1 mm (3 mm)

**Table 3:** Intraoperator repeatability for epithelium thickness obtained using MS-39 in moderate keratoconus patients

Parameter	Observer	Mean ± SD (μm)	S_w_ (μm)	TRT (μm)	CoV (%)	ICC (95% CI)
CET	1st	50.35 ± 5.73	1.31	3.64	2.61	0.949 (0.914 to 0.972)
	2nd	51.25 ± 6.12	1.59	4.41	3.10	0.935 (0.892 to 0.964)
TET	1st	43.20 ± 3.89	1.85	5.12	4.28	0.804 (0.689 to 0.886)
	2nd	43.30 ± 3.51	1.89	5.23	4.36	0.758 (0.627 to 0.857)
S_1_	1st	55.23 ± 5.75	1.29	3.59	2.34	0.951 (0.917 to 0.973)
	2nd	55.47 ± 6.05	1.66	4.59	2.99	0.929 (0.881 to 0.960)
S_3_	1st	51.33 ± 4.42	1.87	5.19	3.65	0.840 (0.736 to 0.911)
	2nd	51.85 ± 5.00	2.37	6.57	4.58	0.804 (0.689 to 0.887)
I_1_	1st	47.95 ± 4.64	1.23	3.41	2.57	0.933 (0.887 to 0.963)
	2nd	48.89 ± 4.74	1.52	4.21	3.11	0.904 (0.841 to 0.946)
I_3_	1st	52.79 ± 5.11	1.62	4.50	3.08	0.905 (0.843 to 0.947)
	2nd	53.26 ± 4.93	1.47	4.06	2.76	0.916 (0.861 to 0.953)
N_1_	1st	52.56 ± 6.53	1.31	3.62	2.48	0.961 (0.934 to 0.979)
	2nd	55.19 ± 5.53	1.21	3.36	2.20	0.953 (0.921 to 0.974)
N_3_	1st	55.27 ± 5.16	1.60	4.42	2.89	0.910 (0.850 to 0.950)
	2nd	56.41 ± 4.58	1.63	4.51	2.88	0.884 (0.809 to 0.934)
T_1_	1st	51.09 ± 5.33	1.50	4.16	2.94	0.925 (0.874 to 0.958)
	2nd	49.88 ± 5.63	1.77	4.91	3.55	0.907 (0.846 to 0.948)
T_3_	1st	54.27 ± 4.01	1.59	4.40	2.93	0.858 (0.769 to 0.919)
	2nd	53.90 ± 4.65	1.38	3.83	2.56	0.917 (0.861 to 0.954)

*SD* = standard deviation; *S*_*w*_ = within-subject standard deviation; *TRT* = test-retest repeatability (2.77 S_w_); *CoV* = coefficient of variation; *ICC* = intraclass correlation coefficient; *CET* = central epithelium thickness; *TET* = thinnest epithelium thickness; *S*_*1*_ *(S*_*3*_*)* = corneal apex superior at 1 mm (3 mm); *I*_*1*_ *(I*_*3*_*)* = corneal apex inferior at 1 mm (3 mm); *N*_*1*_ *(N*_*3*_*)* = corneal apex nasal at 1 mm (3 mm); *T*_*1*_ *(T*_*3*_*)* = corneal apex temporal at 1 mm (3 mm)

**Table 4:** Intraoperator repeatability for epithelium thickness obtained using MS-39 in severe keratoconus patients

Parameter	Observer	Mean ± SD (μm)	S_w_ (μm)	TRT (μm)	CoV (%)	ICC (95% CI)
CET	1st	47.27 ± 5.69	1.90	5.26	4.02	0.896 (0.836 to 0.938)
	2nd	48.09 ± 6.40	1.75	4.84	3.64	0.929 (0.887 to 0.957)
TET	1st	39.96 ± 3.81	2.04	5.65	5.10	0.760 (0.639 to 0.852)
	2nd	40.50 ± 5.59	2.33	6.46	5.76	0.844 (0.759 to 0.905)
S_1_	1st	52.58 ± 8.55	1.87	5.19	3.57	0.953 (0.925 to 0.973)
	2nd	52.74 ± 7.92	2.03	5.61	3.84	0.937 (0.900 to 0.963)
S_3_	1st	53.24 ± 4.84	2.33	6.46	4.38	0.799 (0.692 to 0.878)
	2nd	52.91 ± 4.58	2.74	7.58	5.17	0.711 (0.579 to 0.817)
I_1_	1st	46.74 ± 5.47	2.24	6.21	4.79	0.849 (0.766 to 0.909)
	2nd	47.04 ± 5.42	1.83	5.06	3.88	0.894 (0.835 to 0.936)
I_3_	1st	49.90 ± 6.49	1.53	4.24	3.07	0.946 (0.913 to 0.968)
	2nd	50.28 ± 6.36	1.85	5.12	3.68	0.920 (0.874 to 0.952)
N_1_	1st	49.76 ± 7.17	1.89	5.25	3.81	0.933 (0.893 to 0.961)
	2nd	52.25 ± 6.54	1.98	5.48	3.79	0.914 (0.864 to 0.948)
N_3_	1st	57.05 ± 6.67	2.00	5.53	3.50	0.916 (0.866 to 0.950)
	2nd	57.95 ± 6.06	1.67	4.61	2.87	0.928 (0.886 to 0.957)
T_1_	1st	49.74 ± 5.93	2.14	5.93	4.30	0.880 (0.812 to 0.928)
	2nd	48.69 ± 7.62	2.12	5.88	4.36	0.926 (0.883 to 0.956)
T_3_	1st	55.46 ± 6.38	1.78	4.92	3.20	0.926 (0.882 to 0.956)
	2nd	54.83 ± 5.75	1.87	5.17	3.41	0.902 (0.845 to 0.941)

*SD* = standard deviation; *S*_*w*_ = within-subject standard deviation; *TRT* = test-retest repeatability (2.77 S_w_); *CoV* = coefficient of variation; *ICC* = intraclass correlation coefficient; *CET* = central epithelium thickness; *TET* = thinnest epithelium thickness; *S*_*1*_ *(S*_*3*_*)* = corneal apex superior at 1 mm (3 mm); *I*_*1*_ *(I*_*3*_*)* = corneal apex inferior at 1 mm (3 mm); *N*_*1*_ *(N*_*3*_*)* = corneal apex nasal at 1 mm (3 mm); *T*_*1*_ *(T*_*3*_*)* = corneal apex temporal at 1 mm (3 mm)

**Table 5:** Intraoperator repeatability for epithelium thickness obtained using MS-39 in total patients

Parameter	Observer	Mean ± SD (μm)	S_w_ (μm)	TRT (μm)	CoV (%)	ICC (95% CI)
CET	1st	50.40 ± 5.54	1.34	3.70	2.65	0.944 (0.926 to 0.958)
	2nd	50.97 ± 5.86	1.41	3.90	2.77	0.944 (0.927 to 0.958)
TET	1st	42.88 ± 4.21	1.89	5.23	4.41	0.822 (0.773 to 0.864)
	2nd	43.24 ± 4.70	1.99	5.50	4.60	0.840 (0.796 to 0.878)
S_1_	1st	53.97 ± 6.10	1.40	3.89	2.60	0.949 (0.933 to 0.962)
	2nd	54.06 ± 5.94	1.59	4.40	2.94	0.932 (0.911 to 0.948)
S_3_	1st	51.16 ± 4.63	2.06	5.72	4.04	0.825 (0.773 to 0.868)
	2nd	51.09 ± 4.65	2.34	6.47	4.57	0.784 (0.727 to 0.834)
I_1_	1st	49.18 ± 5.21	1.53	4.24	3.11	0.919 (0.894 to 0.939)
	2nd	49.63 ± 5.34	1.43	3.97	2.89	0.931 (0.911 to 0.948)
I_3_	1st	51.50 ± 5.27	1.61	4.46	3.13	0.912 (0.885 to 0.934)
	2nd	51.78 ± 5.13	1.71	4.72	3.29	0.897 (0.867 to 0.922)
N_1_	1st	52.17 ± 6.25	1.40	3.89	2.69	0.951 (0.936 to 0.963)
	2nd	54.13 ± 5.49	1.46	4.04	2.69	0.933 (0.912 to 0.949)
N_3_	1st	54.93 ± 5.45	1.69	4.68	3.08	0.910 (0.883 to 0.932)
	2nd	55.74 ± 5.09	1.60	4.42	2.86	0.908 (0.880 to 0.930)
T_1_	1st	51.41 ± 5.06	1.55	4.30	3.02	0.911 (0.885 to 0.933)
	2nd	50.42 ± 6.05	1.67	4.63	3.32	0.927 (0.905 to 0.945)
T_3_	1st	53.94 ± 4.79	1.60	4.42	2.96	0.897 (0.866 to 0.922)
	2nd	53.44 ± 4.78	1.56	4.32	2.92	0.901 (0.871 to 0.925)

*SD* = standard deviation; *S*_*w*_ = within-subject standard deviation; *TRT* = test-retest repeatability (2.77 S_w_); *CoV* = coefficient of variation; *ICC* = intraclass correlation coefficient; *CET* = central epithelium thickness; *TET* = thinnest epithelium thickness; *S*_*1*_ *(S*_*3*_*)* = corneal apex superior at 1 mm (3 mm); *I*_*1*_ *(I*_*3*_*)* = corneal apex inferior at 1 mm (3 mm); *N*_*1*_* (N*_*3*_*)* = corneal apex nasal at 1 mm (3 mm); *T*_*1*_ *(T*_*3*_*)* = corneal apex temporal at 1 mm (3 mm)

### Interobserver reproducibility in the measurement of ET

The reproducibility of ET measurement in the FFKC group, mild KC group, moderate KC group, severe KC group, and the total subject is listed in Additional file 1: Tables S1–S5, including CET, TET, S_1_, S_3_, I_1_, I_3_, N_1_, N_3_, T_1_, T_3_. In all groups, N_1_ always shows the maximum TRT and CoV values: FFKC 4.95 μm, 3.31%; mild KC 7.65 μm, 5.09%; moderate KC 9.62 μm, 6.42%; severe KC 10.00 μm, 7.05%; total 8.59 μm, 5.82%. Concerning ICCs, almost all the values are more than 0.73 in the above group, except for the N_1_, TET of severe KC. Figure 3 presents TRT values of reproducibility at different stages of KC patients at all locations in the form of histograms.

## Discussion

The Global Consensus on Keratoconus and Ectatic Diseases in 2015 suggested that an increased curvature of the anterior or posterior corneal surfaces and/or a thinning of the cornea were suggestive of keratoconus disease progression [2]. However, there is no strict definition of keratoconus disease progression, although K_max_ is the most frequently used parameter. However, ET measurement is an intriguing parameter for assessing keratoconus disease progression [12]. Epithelial cell activation and regrowth are related to the biomechanics of the cornea [18], especially to corneal thinning, which is closely related to keratoconus progression [28]. In addition, the MS-39 has high repeatability in measurements of the ET in patients with keratoconus but also in post-refractive surgery ectasia and in healthy subjects [29–31]. Despite the fact that corneal biologic properties change with severity, the repeatability and reproducibility of MS-39 in KC of varying severity have not been studied [32]. This factor is crucial because earlier studies have shown that it is necessary to take the severity of the keratoconus disease into account. The findings of this investigation suggest the differences in the precision of keratoconus epithelial measurements to determine the cut-offs of the progression of different degrees of keratoconus.

### Precision in the measurement of ET

Except for S_1_, ET at the 1 mm location tended to decrease with increasing KC severity. This trend is more prominent in both CET and TET. Previous studies have shown that the values of TET decrease gradually in the order of the control group (53.4 ± 3.3 μm), FFKC (49.7 ± 2.9 μm), moderate KC (46.6 ± 5.4 μm), and severe KC (46.3 ± 6.4 μm) groups [33]. In our study, ETs generally showed a gradual thinning trend as severity increased in CET, TET, I_1_, N_1_, and T_1_. Toprak et al. found that the TET value of FFKC group (47.3 ± 3.8 μm) was significantly lower than the control group (48.5 ± 2.9 μm) [34], but there was no significant difference between CET. The TET of FFKC group in the current study, however, was significantly thicker when compared to clinical KC groups. To diagnose and predict the progression of early KC, TET may be a more sensitive indicator than other parameters.

The TRT values of the measuring points at 3 mm (4.42 to 5.74 μm) was consistently greater than those at 1 mm (3.89 to 4.3 μm) in all subjects. In comparison to the inferior, the superior's repeatability was worse, especially at the 3 mm measurement point, which was likely caused by inadequate eye exposure or eyelash occlusion. In subgroups, FFKC and mild KC showed the same trend but gradually became unpredictable as the severity of KC increased. Besides, at points of 1 mm, TRT values were found to be gradually larger with the increase of KC severity in all groups. Repeatability differences are not as apparent at 3 mm points. This may be due to the cone position which is mostly close to the central region of the cornea. According to TRT, CET and TET also revealed the tendency of decreased repeatability with increased severity of KC. Lu et al. divided KC into groups of mild and advanced cases and evaluated the repeatability of RTVue, which revealed similar trends to those observed in earlier studies (Optovue, Inc. Fremont, CA) [35–37]. The repeatability of the central area seemed better than the peripheral as demonstrated by TRT: 3.91 and 6.7 µm in the central region of mild and advanced KC, 2.83 to 7.98 µm and 4.65 to 11.11 µm in the other regions. Furthermore, the measurement of ET became unstable as keratoconus progressed, with the TRT ranging from 3.77 to 7.98 µm in mild KC and 5.32 to 11.11 µm in advanced KC. Similar rules could also be found in our study. The epithelium of KC patients undergo the following histological changes, especially in the more severe cases: wing cells display large and irregularly shaped nuclei, epithelial cells assume an elongated shape, as well as the disruption of Bowman’s layer, etc. [38]. However, the changes in Bowman’s layer make it a challenge for optical coherence tomography (OCT) to correctly demarcate the epithelial boundary [39]. This is perhaps why repeatability is reduced in more severe KC. Our study found that MS-39 had acceptable repeatability in measuring KC. The total group, nevertheless, tended to have higher TRT than the mild group and lower TRT than the severe group even though TRTs were not the same at the various stages of KC. To prevent errors in judgment in the repeatability analysis of KC patients, this indicates that hierarchical discussion must be conducted.

The TRT values of the nasal and temporal show noticeably higher values in the reproducibility study when compared to other quadrants. Unexpectedly, the TRTs at the nasal and temporal points at 1 mm are higher than those at 3 mm regardless of the stage of KC. Georgeon et al. [40] studied the reproducibility of ET provided by MS-39 in normal eyes and found that the superior regions usually showed poor reproducibility, which differed from the findings in our study. The altered morphology of the KC could lead to lower reproducibility. Sella et al. [36] used an iVue device (Optovue, Inc. Fermont, CA) to measure ET in KC patients, and gained the CoV results (range: 2.2% to 4.1%) of reproducibility which was similar to our study, except for N_1_, T_1_. Ma et al. [41] divided the cornea into four regions by RTVue: 2 mm diameter central zone, 2 to 5 mm diameter paracentral zones, 5 to 7 mm diameter mid-peripheral zones, 7 to 9 mm diameter peripheral zones, and the values of nasal and temporal S_w_ obtained (range: 1.4 to 2.1 µm) were lower than the corresponding points in our study. The TRT values of the nasal and temporal regions in our study differ from others. This may be due to the cone position in KC patients which can affect measurement results. Therefore, it is essential to determine the cone of ET. The rule that TRT values increased with severity is still in place even though nasal and temporal reproducibility decreased.

### Progression cut-offs judgement of TET and CET

Repeatability limits can be used to determine whether changes in a parameter or inaccuracies in measurement was indeed the real cause. Epithelial measurements have recently been demonstrated to be useful in identifying eyes with KC that is actively progressing. TET and CET have also been repeatedly demonstrated to be effective in the diagnosis of keratoconus [19]. Temstet et al. [33] and Toprak et al. [41] both found out that TET may be a sensitive indicator for early keratoconus diagnosis. Furthermore, our study clearly demonstrated the distinction between TET and CET at various stages of KC. The precise measurement of TET and CET appears to be particularly crucial. Based on multiple measurements by two observers, we consider that FFKC is likely to occur when TET is less than 46.28 µm or CET is less than 52.79 µm. The mean difference of TET between the control and FFKC group was 3.7 µm in the study of Temstet et al. [33] Yang et al. [42] reported the mean difference of 3.04 µm when comparing the control group and the FFKC group. This means when the measurement error is greater than 3 µm, there is a possibility of clinical misdiagnosis in early KC diagnosis. Georgeon et al. [40] proposed that MS-39 has high reproducibility in the measurement of TET for healthy people, with the S_w_ value being 1.18 µm and the corresponding TRT (2.77S_w_) was 3.27 µm. Li et al. [43] obtained an acceptable S_w_ value of 1.8 µm (TRT 4.97 µm) when evaluating the repeatability measurement for TET of KC. However, the TRTs of TET ranged between 2.22 and 7.42 µm in our study, depending on disease severity. The single cut-off applied to all patients tends to overestimate severe cases and undervalue milder ones. Consequently, we chose 4.9 µm, 5.2 µm, and 7.4 µm as the cut-off values for mild KC, moderate KC, and severe KC, respectively. TRTs above those thresholds were regarded as unacceptable for repeatability or reproducibility. In terms of CET, the cut-off values for mild KC, moderate KC, and severe KC would be 2.8 µm, 4.4 µm, 5.3 µm, respectively. Previous studies have confirmed that in patients with mild KC (K_max_ < 48.00 D), a change in K_max_ of only 0.50 D is required to determine progression, and in severe patients (K_max_ > 58.00 D) an increase in K_max_ to 1.50 D is required to determine progression. For TCT, a reduction of 7 μm in mild KC and 11 μm in severe KC determines progression using MS-39. Because of various challenges, values are still not clearly defined, and it is still difficult to assess KC progress. Nevertheless, the epithelium can be used as a meaningful parameter for KC progression assessment. We demonstrate that the precision of ET measurement by MS-39 decreased as keratoconus severity increased but TET of FFKC and mild KC group. This may be due to insignificant changes in the epithelium of the thinnest point keratoconus at the early stages. However, it is advisable to refer to general trends in clinical practice instead of precise values from our current study. After analysis, we attribute this to the irregularity of keratoconus, which would increase the uncertainty and difficulty of measurement. Moreover, the measurement error increases as the measuring point is further away from the corneal apex in our study, which is consistent with other studies [40, 42]. This may be because the incident angle between the OCT probe beam and the corneal surface increases as the distance from the center of the cornea increases [40].

Our work has limitations. Epithelial, stromal, and total corneal thickness profiles are critical for other corneal conditions, such as post-LASIK keratectasia and pellucid marginal degeneration, which were not considered in this study. In future studies, more metrics should be included, and a broader range of corneal pathologies should be examined. In addition, other principles of measurement were not compared in this study.

## Conclusions

MS-39 demonstrated acceptable repeatability and reproducibility in measuring patients with KC. However, reproducibility and repeatability would decrease gradually with the peripheralization of the measurement points and the severity of KC. When assessing epithelial corneal thickness, MS-39 should be used with caution to check for corneal ectasia or track the development of keratoconus. The repeatability coefficients of KC cannot be seen uniformly due to the measurement variability of the instrument. It is necessary to set up stratified progression thickness limits based on the severity of the disease; for TET, significant progress is defined as decreases of 4.9 µm in mild KC, 5.2 µm in moderate KC, and 7.4 µm in severe KC.

### Supplementary Information

**Additional file 1.** Interobserver reproducibility in the measurement of corneal epithelium thickness.

## Data Availability

All data generated or analyzed during this study are included in this published article.

## Abbreviations

Kmax: Maximum keratometry value
I-S value: Inferior-superior difference value
KISA% index: Keratoconus percentage index
TKC: Topographical keratoconus classification
ISV: Index of surface variance
KI: Keratoconus index
Rmin: Smallest radius
SLED: Super luminescent light-emitting diode
CET: Center epithelium thickness
TET: Thinnest epithelium thickness
SD: Standard deviation
S_w_: Within-subject standard deviation
TRT: Test–retest variability
CoV: Coefficient of variation
ICC: Intraclass correlation coefficient
