# Supplementary material for: Assessing progression limits in different grades of keratoconus from a novel perspective: precision of measurements of the corneal epithelium
Source: Eye Vis (Lond). 2024 Jan 2;11:1. doi: 10.1186/s40662-023-00368-9 (PMC10759576; doi:10.1186/s40662-023-00368-9)
Supplement: Supplementary file 1 — Additional file 1. Interobserver reproducibility in the measurement of corneal epithelium thickness. [file 40662_2023_368_MOESM1_ESM.docx]

| Table S1. Interobserver reproducibility for epithelium thickness obtained using MS-39 in forme fruste keratoconus patients. | | | | | | |
| --- | --- | --- | --- | --- | --- | --- |
| Parameter | Mean ± SD (μm) | S_w_ (μm) | TRT (μm) | COV (%) | ICC (95% CI) |  |
| CET | 52.79 ± 2.91 | 0.57 | 1.59 | 1.09 | 0.960 (0.916 to 0.981) |  |
| TET | 46.28 ± 3.6 | 0.80 | 2.22 | 1.73 | 0.950 (0.895 to 0.977) |  |
| S_1_ | 53.47 ± 2.44 | 0.55 | 1.53 | 1.03 | 0.947 (0.888 to 0.975) |  |
| S_3_ | 49.73 ± 3.12 | 1.04 | 2.88 | 2.09 | 0.898 (0.774 to 0.955) |  |
| I_1_ | 52.71 ± 2.83 | 0.60 | 1.66 | 1.14 | 0.954 (0.904 to 0.979) |  |
| I_3_ | 52.07 ± 3.28 | 1.14 | 3.15 | 2.18 | 0.882 (0.762 to 0.944) |  |
| N_1_ | 53.94 ± 2.84 | 1.79 | 4.95 | 3.31 | 0.662 (0.389 to 0.828) |  |
| N_3_ | 53.52 ± 3.20 | 1.38 | 3.83 | 2.58 | 0.813 (0.635 to 0.909) |  |
| T_1_ | 52.76 ± 3.30 | 1.38 | 3.83 | 2.62 | 0.830 (0.665 to 0.917) |  |
| T_3_ | 52.66 ± 2.97 | 1.35 | 3.73 | 2.55 | 0.808 (0.626 to 0.906) |  |
| SD = standard deviation; S_w_ = within-subject standard deviation; TRT = test-retest repeatability (2.77 S_w_); ICC = intraclass correlation coefficient; CET = central epithelium thickness; TET = thinnest epithelium thickness; S_1_ (S_3_) = corneal apex superior at 1 mm (3 mm); I_1_ (I_3_) = corneal apex inferior at 1 mm (3 mm); N_1_ (N_3_) = corneal apex nasal at 1 mm (3 mm); T_1_ (T_3_) = corneal apex temporal at 1 mm (3 mm) | | | | | |  |

| Table S2. Interobserver reproducibility for epithelium thickness obtained using MS-39 in mild keratoconus patients. | | | | | | |
| --- | --- | --- | --- | --- | --- | --- |
| Parameter | Mean ± SD (μm) | S_w_ (μm) | TRT (μm) | COV (%) | ICC (95% CI) |  |
| CET | 52.48 ± 4.54 | 0.85 | 2.36 | 1.63 | 0.968 (0.934 to 0.985) |  |
| TET | 43.98 ± 3.08 | 1.27 | 3.52 | 2.89 | 0.834 (0.680 to 0.917) |  |
| S_1_ | 54.88 ± 3.99 | 1.28 | 3.54 | 2.33 | 0.910 (0.819 to 0.956) |  |
| S_3_ | 48.45 ± 3.82 | 1.53 | 4.24 | 3.16 | 0.875 (0.733 to 0.944) |  |
| I_1_ | 50.62 ± 4.15 | 0.99 | 2.75 | 1.96 | 0.948 (0.895 to 0.975) |  |
| I_3_ | 51.96 ± 4.36 | 1.05 | 2.91 | 2.02 | 0.943 (0.885 to 0.973) |  |
| N_1_ | 54.21 ± 4.41 | 2.76 | 7.65 | 5.09 | 0.688 (0.440 to 0.838) |  |
| N_3_ | 53.20 ± 3.81 | 2.01 | 5.57 | 3.78 | 0.764 (0.562 to 0.880) |  |
| T_1_ | 51.98 ± 4.09 | 2.32 | 6.43 | 4.47 | 0.736 (0.516 to 0.865) |  |
| T_3_ | 52.16 ± 3.74 | 1.38 | 3.82 | 2.64 | 0.876 (0.757 to 0.939) |  |
| SD = standard deviation; S_w_ = within-subject standard deviation; TRT = test-retest repeatability (2.77 S_w_); ICC = intraclass correlation coefficient; CET = central epithelium thickness; TET = thinnest epithelium thickness; S_1_ (S_3_) = corneal apex superior at 1 mm (3 mm); I_1_ (I_3_) = corneal apex inferior at 1 mm (3 mm); N_1_ (N_3_) = corneal apex nasal at 1 mm (3 mm); T_1_ (T_3_) = corneal apex temporal at 1 mm (3 mm) | | | | | |  |

| Table S3. Interobserver reproducibility for epithelium thickness obtained using MS-39 in moderate keratoconus patients. | | | | | | |
| --- | --- | --- | --- | --- | --- | --- |
| Parameter | Mean ± SD (μm) | S_w_ (μm) | TRT (μm) | COV (%) | ICC (95% CI) |  |
| CET | 51.16 ± 6.02 | 0.95 | 2.64 | 1.86 | 0.975 (0.951 to 0.987) |  |
| TET | 43.18 ± 3.59 | 1.51 | 4.17 | 3.49 | 0.837 (0.703 to 0.913) |  |
| S_1_ | 55.50 ± 5.66 | 1.64 | 4.54 | 2.96 | 0.924 (0.856 to 0.96) |  |
| S_3_ | 51.83 ± 4.63 | 1.73 | 4.80 | 3.34 | 0.872 (0.757 to 0.935) |  |
| I_1_ | 48.57 ± 4.73 | 0.97 | 2.68 | 1.99 | 0.959 (0.920 to 0.979) |  |
| I_3_ | 52.98 ± 4.83 | 1.30 | 3.60 | 2.45 | 0.934 (0.874 to 0.966) |  |
| N_1_ | 54.12 ± 5.58 | 3.47 | 9.62 | 6.42 | 0.685 (0.463 to 0.826) |  |
| N_3_ | 55.85 ± 4.57 | 2.04 | 5.64 | 3.65 | 0.828 (0.688 to 0.908) |  |
| T_1_ | 50.78 ± 5.26 | 2.78 | 7.71 | 5.48 | 0.744 (0.554 to 0.861) |  |
| T_3_ | 53.97 ± 4.03 | 1.88 | 5.22 | 3.49 | 0.812 (0.662 to 0.899) |  |
| SD = standard deviation; S_w_ = within-subject standard deviation; TRT = test-retest repeatability (2.77 S_w_); ICC = intraclass correlation coefficient; CET = central epithelium thickness; TET = thinnest epithelium thickness; S1 (S3) = corneal apex superior at 1 mm (3 mm); I1 (I3) = corneal apex inferior at 1 mm ( 3mm); N1 (N3) = corneal apex nasal at 1 mm (3 mm); T1 (T3) = corneal apex temporal at 1 mm (3 mm) | | | | | |  |

| Table S4. Interobserver reproducibility for epithelium thickness obtained using MS-39 in severe keratoconus patients. | | | | | | |
| --- | --- | --- | --- | --- | --- | --- |
| Parameter | Mean ± SD (μm) | S_w_ (μm) | TRT (μm) | COV (%) | ICC (95% CI) |  |
| CET | 47.86 ± 6.03 | 1.70 | 4.71 | 3.56 | 0.923 (0.863 to 0.957) |  |
| TET | 40.03 ± 4.53 | 2.68 | 7.42 | 6.69 | 0.691 (0.496 to 0.820) |  |
| S_1_ | 52.76 ± 8.09 | 1.73 | 4.78 | 3.27 | 0.957 (0.923 to 0.976) |  |
| S_3_ | 53.14 ± 4.43 | 1.97 | 5.47 | 3.71 | 0.830 (0.706 to 0.905) |  |
| I_1_ | 46.93 ± 5.37 | 1.38 | 3.83 | 2.94 | 0.937 (0.888 to 0.965) |  |
| I_3_ | 49.96 ± 6.3 | 1.78 | 4.94 | 3.57 | 0.925 (0.866 to 0.958) |  |
| N_1_ | 51.25 ± 6.58 | 3.61 | 10.00 | 7.05 | 0.737 (0.565 to 0.847) |  |
| N_3_ | 57.51 ± 6.05 | 2.64 | 7.33 | 4.60 | 0.832 (0.712 to 0.905) |  |
| T_1_ | 49.33 ± 6.48 | 3.24 | 8.98 | 6.57 | 0.782 (0.633 to 0.875) |  |
| T_3_ | 55.30 ± 5.76 | 2.81 | 7.79 | 5.09 | 0.788 (0.643 to 0.878) |  |
| SD = standard deviation; S_w_ = within-subject standard deviation; TRT = test-retest repeatability (2.77 S_w_); ICC = intraclass correlation coefficient; CET = central epithelium thickness; TET = thinnest epithelium thickness; S_1_ (S_3_) = corneal apex superior at 1 mm (3 mm); I_1_ (I_3_) = corneal apex inferior at 1 mm (3 mm); N_1_ (N_3_) = corneal apex nasal at 1 mm (3 mm); T_1_ (T_3_) = corneal apex temporal at 1 mm (3 mm) | | | | | |  |

| Table S5. Interobserver reproducibility for epithelium thickness obtained using MS-39 in total patients. | | | | | | |
| --- | --- | --- | --- | --- | --- | --- |
| Parameter | Mean ± SD (μm) | S_w_ (μm) | TRT (μm) | COV (%) | ICC (95% CI) |  |
| CET | 50.88 ± 5.65 | 1.23 | 3.41 | 2.42 | 0.954 (0.937 to 0.967) |  |
| TET | 43.03 ± 4.39 | 2.00 | 5.54 | 4.65 | 0.801 (0.733 to 0.854) |  |
| S_1_ | 54.14 ± 5.84 | 1.45 | 4.02 | 2.68 | 0.943 (0.921 to 0.959) |  |
| S_3_ | 51.13 ± 4.50 | 1.68 | 4.66 | 3.29 | 0.877 (0.829 to 0.912) |  |
| I_1_ | 49.51 ± 5.21 | 1.11 | 3.07 | 2.24 | 0.957 (0.940 to 0.969) |  |
| I_3_ | 51.66 ± 5.10 | 1.39 | 3.85 | 2.69 | 0.930 (0.903 to 0.949) |  |
| N_1_ | 53.34 ± 5.51 | 3.10 | 8.59 | 5.82 | 0.732 (0.644 to 0.800) |  |
| N_3_ | 55.36 ± 5.00 | 2.13 | 5.91 | 3.85 | 0.839 (0.782 to 0.882) |  |
| T_1_ | 51.09 ± 5.29 | 2.64 | 7.30 | 5.16 | 0.781 (0.707 to 0.838) |  |
| T_3_ | 53.76 ± 4.55 | 2.04 | 5.66 | 3.80 | 0.820 (0.757 to 0.867) |  |
| SD = standard deviation; S_w_ = within-subject standard deviation; TRT = test-retest repeatability (2.77 S_w_); ICC = intraclass correlation coefficient; CET = central epithelium thickness; TET = thinnest epithelium thickness; S_1_ (S_3_) = corneal apex superior at 1 mm (3 mm); I_1_ (I_3_) = corneal apex inferior at 1 mm (3 mm); N_1_ (N_3_) = corneal apex nasal at 1 mm (3 mm); T_1_ (T_3_) = corneal apex temporal at 1 mm (3mm) | | | | | |  |
